# The synergistic influence of polyethyleneimine-grafted graphene oxide and iodide for the protection of steel in acidizing conditions

**DOI:** 10.1039/d0ra00864h

**Published:** 2020-05-06

**Authors:** K. R. Ansari, Dheeraj Singh Chauhan, M. A. Quraishi, A. Y. Adesina, Tawfik A. Saleh

**Affiliations:** Center of Research Excellence in Corrosion, Research Institute, King Fahd University of Petroleum and Minerals Dhahran 31261 Saudi Arabia mumtaz.quraishi@kfupm.edu.sa +966538600057; Chemistry Department, King Fahd University of Petroleum and Minerals Dhahran 31261 Saudi Arabia tawfik@kfupm.edu.sa

## Abstract

Herein, graphene oxide (GO) was chemically functionalized with polyethyleneimine (PEI) in a single step to obtain PEI-GO, which was characterized *via* FTIR spectroscopy, SEM, and TEM. Additionally, for the first time, PEI-GO was employed for the corrosion mitigation of carbon steel in a solution of 15% HCl. The corrosion performance of the inhibitor was evaluated by utilizing weight loss tests, electrochemical measurements with impedance analysis, electrochemical frequency modulation, and potentiodynamic polarization studies. Thorough surface analysis was performed using 3D profilometry and static water contact angle measurements. PEI-GO was adsorbed on the steel surface and showed mixed-type corrosion inhibition behavior with the prevalence of cathodic characteristics. Additionally, potassium iodide was incorporated in the acid solution as a synergistic agent to enhance the corrosion inhibition behavior of PEI-GO. The obtained results showed that PEI-GO alone provided a high corrosion inhibition efficiency of 88.24% at a temperature of 65 °C and in the presence of KI, it showed an I.E. of 95.77% due to their synergistic effect. These interesting results demonstrate that PEI-GO can act as a potential corrosion inhibitor in acidizing conditions. The DFT-based computational studies showed that the inhibitor functioned in both its neutral and protonated forms.

## Abstract 2

Herein, graphene oxide (GO) was chemically functionalized with polyethyleneimine (PEI) in a single step to obtain PEI-GO, which was characterized *via* FTIR spectroscopy, SEM, and TEM.

## Graphical abstract

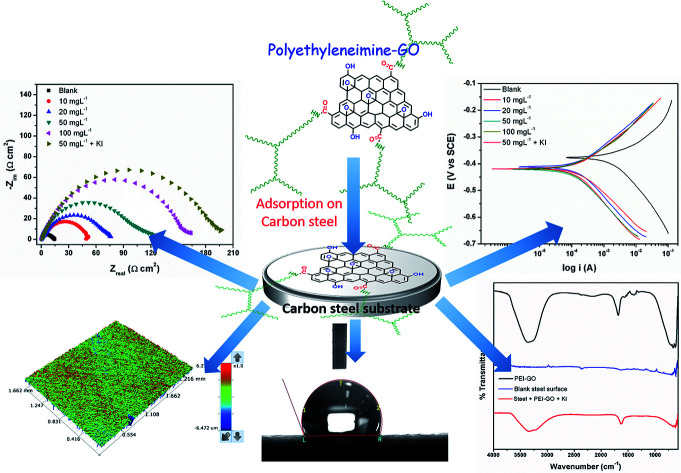

## 1 Introduction

Graphene has potential applications in the development of anticorrosion coatings due to its nanosize and hydrophobicity, which promote its appeal as a barrier coating.^1–4^ Thus, graphene is used as a nanofiller in composite coatings to reduce the porosity and improve the hydrophobicity of the coatings.^5–8^ However, the practical utility of graphene in corrosion inhibition is restricted due to its limited aqueous solubility. In contrast, graphene oxide (GO) is a considerably hydrophilic material that shows superior aqueous solubility owing to its oxygen-based surface functionalities, which improve its dispersibility in aqueous solutions. Gupta *et al.*, for the first time, used chemically functionalized GO as a corrosion inhibitor for steel solutions in acid environments.^9^ Subsequently, in recent years, a few reports have appeared in the literature on the use of pyridine, aminobenzene, aminophenol, *etc.* functionalized GO in the corrosion inhibition of steel in an acidic environment.^9–12^

Carbon steel has a plethora of applications in several industries, such as in the oil–gas and petroleum sectors.^13,14^ These applications involve the use of high concentrations of corrosive mineral acids such as hydrochloric acid and sulphuric acid.^15–17^ However, these acids cause severe corrosion of steel structures and lead to immense economic loss and potential hazards to human life. Accordingly, to protect and prolong the life of steel structures, it is a common practice to use organic compounds as corrosion inhibitors.^16–23^ These organic inhibitors are mainly nitrogen-containing organic compounds such as azoles, imidazolines, pyridines, and pyrimidines.^24–26^ However, the synthesis of these organic compounds requires long synthetic steps, and the requirement of high concentrations in strongly acidic environments increases the cost of their application. Therefore, scientists worldwide have directed their efforts towards the exploration of novel and environmentally benign corrosion inhibitors.

The remarkable behavior of chemically modified GO directed our attention towards the exploration of polyethylene-imine (PEI)-modified GO (PEI-GO) in the corrosion inhibition of carbon steel in a 15% HCl medium. PEI-GO has been reported as a nanocarrier for drug and gene delivery due to its considerably low-toxic nature.^27,28^ Herein, for the first time, we report the use of PEI-GO as a corrosion inhibitor in oil-well acidizing applications. Considering the high temperatures used in industrial processes, we studied the effect of temperature, ranging from ambient to high temperature (35 °C to 65 °C), in the present work. Further, potassium iodide was also incorporated in the corrosive solution to induce a synergistic improvement in the corrosion inhibition of PEI-GO. A detailed study was performed employing chemical and electrochemical measurements supported with surface analysis *via* FTIR, 3D profilometry, and contact angle measurements.

## 2 Experimental

### 2.1 Synthesis of corrosion inhibitor

**Fig. 1:**
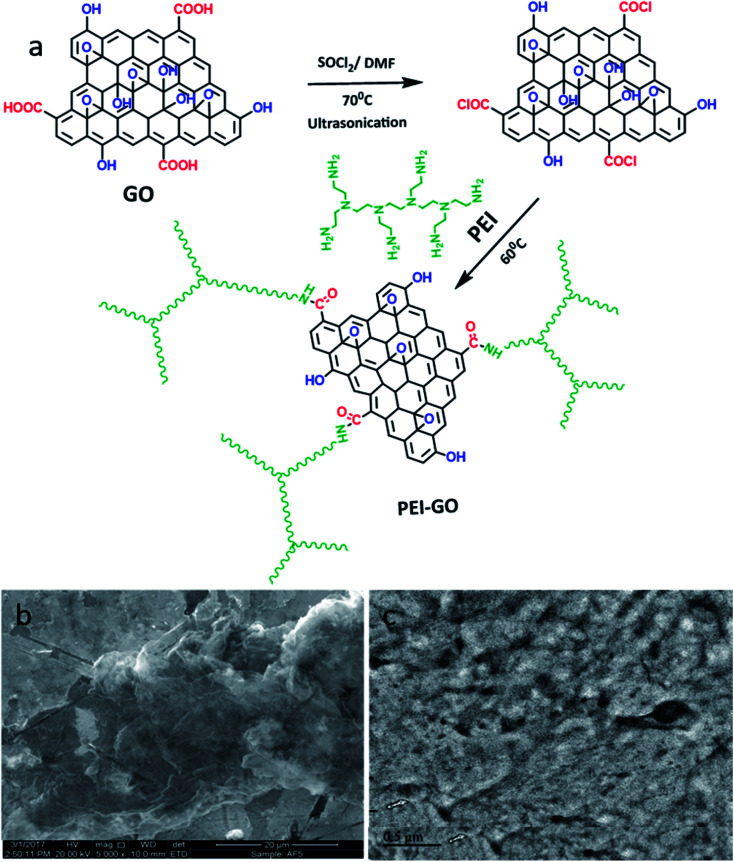
(a) Schematic presentation of the synthesis of PEI-GO. TEM images of the synthesized (b) GO and (c) PEI-GO.

Polyethyleneimine and other chemicals were procured from Sigma Aldrich. Hydrochloric acid (Sigma Aldrich 37%) was used for preparing the corrodent solutions. Graphene oxide was prepared following a modified Hummers' method. The carboxyl groups on graphene were altered to formyl chloride *via* a reaction with thionyl chloride (SOCl_2_), as shown in Fig. 1(a). Briefly, graphene was dispersed in SOCl_2_ in the presence of anhydrous DMF, and this mixture was subjected to ultrasonication for 1 h. Subsequently, the mixture was heated at 80 °C with stirring overnight. Then, the mixture was left to cool, and then the solution was repeatedly rinsed with anhydrous THF. Polyethylenimine-graphene was synthesized as follows. A polyethylenimine solution was added to the Cl-modified graphene. The mixture was stirred at 150 rpm at 90 °C for 24 h and then centrifuged to separate the adsorbent. The TEM images of GO (Fig. 1(b)) show the formation of graphene nanosheets, whereas the TEM images of PEI-GO (Fig. 1(c)) reveal the successful grafting of polymeric PEI chains on the nanosheets of graphene.

### 2.2 Corrosion evaluation

#### 2.2.1 Gravimetric measurements

Carbon steel strips were initially abraded using emery paper, degreased with acetone and washed with distilled water. The cleaned steel samples were stored in a desiccator prior to use. The polished metallic specimens with a size of 2.5 × 2.0 × 0.5 cm were dipped in 15% HCl in the absence and presence of PEI-GO at the desired concentration for 24 h and weighed accurately after the cleaning and drying steps. Following replicate tests, the average weight loss value was measured to obtain the corrosion rate (mm per year) as follows:^26,29,30^1
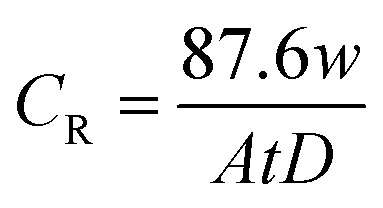
where *w* is the average weight loss in g, *A* is the surface area of the steel sample (cm^−2^), *t* is the immersion period in h, and *D* denotes the carbon steel density in g cm^−3^. The efficiency of inhibition (*η*%) and the surface coverage (*θ*) were acquired from *C*_R_ using the following expressions:^29–32^2
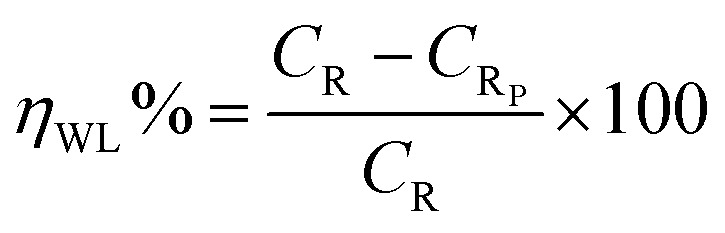
3
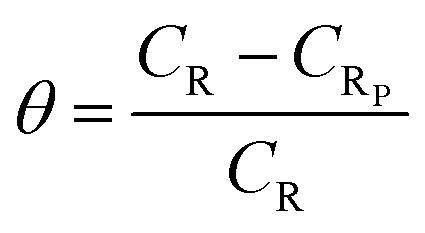
where *C*_R_ and *C*_R_P__ denote the corrosion rate achieved in the absence and presence of the inhibitor PEI-GO, respectively (Table 1).

**Table 1:** Weight loss data for the carbon steel samples after immersion in different concentrations of PEI-GO and with KI

Component	Inhibitor (mg L^−1^)	KI (mM)	Corrosion rates (mm per year)	Coverage (*θ*)	*η*%
Blank	—	—	85.29	—	—
PEI-GO	5	—	19.74	0.77	76.85
	10	—	17.05	0.80	80.01
	25	—	9.82	0.88	88.49
	50	—	8.74	0.89	89.75
	100	—	8.13	0.90	90.46
	—	0.5	61.64	0.28	27.73
	—	1	58.55	0.31	31.34
	—	5	22.14	0.74	74.03
	50	0.5	8.05	0.89	89.91
	50	1	6.28	0.92	91.67
	50	5	3.59	0.96	95.77

#### 2.2.2 Electrochemical corrosion tests

The electrochemical corrosion measurements were performed on a standard three-electrode-based electrochemical cell assembly. A carbon steel strip with a surface area of 1 cm^2^ was employed as the working electrode, the reference electrode was a saturated calomel electrode (SCE), and a cylindrical graphite rod was used as the counter electrode. All the potentials are reported with respect to the SCE. A Gamry Reference 600 electrochemical workstation equipped with the Echem Analyst 6.33 data analysis and interpretation software suite was used for the corrosion tests. All the experiments were conducted in an unstirred and aerated electrochemical cell. After connecting the three electrodes, the steady-state open circuit potential (*E*_OCP_) was run to attain a stable value, after which the different electrochemical measurements were conducted.^33,34^ The EIS measurements were conducted in the frequency range of 100 kHz to 10 mHz at an amplitude of 10 mV. The electrochemical frequency modulation (EFM) experiments were performed using a base frequency of 1 Hz, using 2 Hz and 5 Hz as the perturbation frequencies and 10 mV amplitude. The potentiodynamic polarization measurements (PDP) were performed in the range of ±250 mV *vs.* OCP at a scan rate of 1 mV s^−1^. A fresh working electrode and an electrolyte of 15% HCl were utilized for the electrochemical measurements. The electrochemical tests were conducted in triplicate for the validation of the tests.

### 2.3 Surface analysis

The surface morphology of the carbon steel samples was recorded using a 3D optical profilometer (Contour GT-K, Bruker Nano GmBH, Germany) for visual inspection of the protective influence of the inhibitor film. The metallic specimens were immersed in an acid solution (15% HCl) without and with the optimum inhibitor concentration. After immersion for 24 h, the samples were cleaned, dried, and their surface topography and pit depth analyzed employing the 3D optical profilometer. The water contact angle (WCA) of the mild steel surface after immersion for 24 h in 15% HCl was measured using a contact angle measurement device (VCA OPTIMA, AST Products Inc. USA). The WCA was measured from five different locations on the sample and the mean value presented. A drop of water with a volume of 3 μL was used for the determination of the WCA of the samples, while the sessile drop was fitted using the VCA analysis software (VCA OPTIMA, AST Products Inc. USA). The WCA was measured with a precision of ± 0.1°. FTIR studies were also performed to examine the PEI-GO inhibitor film formed on the carbon steel surface. The attenuated total reflection (ATR) technique was employed using a Nicolet iS5 FTIR.

### 2.4 Quantum chemical study

Density functional theory (DFT) is the most commonly used technique for predicting the chemical reactivity of inhibitor molecules. In the present case, all quantum chemical studies were carried out with DFT/B3LYP methods using the 6-31G basis set in the Gaussian 09 program package.^35^ The quantum chemical parameters associated with the energies, including highest occupied molecular orbital (*E*_HOMO_) and lowest unoccupied molecular orbital (*E*_LUMO_), and the energy gap (Δ*E* = *E*_LUMO_ − *E*_HOMO_) were calculated.

## 3 Results and discussion

### 3.1 Weight loss measurements

#### 3.1.1 Effect of inhibitor dosage

The influence of different concentrations of PEI-GO on the weight loss of the carbon steel coupons was evaluated by administering different dosages of the inhibitor in the range of 5 to 100 mg L^−1^. The results revealed that the inhibition efficacy increased with an increase in the inhibitor concentration and reached 88.24% at a concentration of 50 mg L^−1^, beyond which no significant increase was observed. Herein, it is noteworthy to mention that even at a concentration as low as 5 mg L^−1^, the inhibitor showed a significant efficiency of 76.85%. The steady increase in the efficiency with an increase in the concentration of PEI-GO shows that the inhibitor was adsorbed on the metal surface,^21,36^ and covered the active sites favoring corrosion damage, resulting in a decrease in the rate of corrosion.

The influence of iodide ions in enhancing the inhibition efficiency was examined by administering different concentrations of KI (1 mM to 10 mM) in the presence of the optimum inhibitor concentration (50 mg L^−1^). The inhibition efficiency increased further and reached 95.77% in the presence of 5 mM KI. This suggests that the iodide ions play a key role in enhancing the surface coverage of the PEI-GO molecules. The halide ions bearing a negative charge can adsorb on the surface of the steel, which carries a positive charge due to the continuous loss of electrons on account of corrosive electrodissolution. The iodide ions adsorb on the surface of the metal and promote a coulombic interaction between the positively charged metallic substrate and the positively charged inhibitor molecules (*e.g.*, the free –NH_2_ groups in the present case), thus facilitating the adsorption of PEI-GO on the steel surface.^21,37,38^

#### 3.1.2 Effect of temperature

In the petroleum industry, the oil-well acidizing process is carried out in the bottom-hole tubing, where high temperatures exist. Therefore, to appropriately simulate the acidizing environment practically encountered, we investigated the influence of an increase in temperature from 35 °C to 65 °C during corrosion testing. Generally, an increase in the immersion temperature can cause sorption of PEI-GO from the target steel surface, which increases the corrosion rate and lowers the inhibition effectiveness.^30,31,36^ However, in the present study, it was observed that an increase in temperature did not affect the inhibition efficiency, which can be attributed to the chemical interaction between the metal and the inhibitor. Besides, the addition of KI again resulted in a higher corrosion inhibition efficiency compared to that with PEI-GO alone at elevated temperature. The activation energy (*E*_a_) of the carbon steel surface in the presence of the inhibitor in 15% HCl can be calculated as follows:^39^4
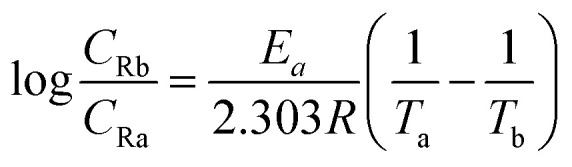
where *C*_Ra_ and *C*_Rb_ represent the corrosion rate and *θ*_a_ and *θ*_b_ represent the surface coverage values of the inhibitor obtained at two temperatures (*T*_a_ and *T*_b_), respectively. The observed value of *E*_a_ in the presence of the inhibitor (63.53 kJ mol^−1^) was higher than that observed in its absence (47.82 kJ mol^−1^). This supports the formation of a film of inhibitor molecules on the steel surface, which acts as a protective barrier and increases the *E*_a_ as a barrier for the corrosion reaction to proceed.^21,30,40^ The addition of KI to the corrosive solution produced a further increase in the *E*_a_ up to 72.63 kJ mol^−1^, which supports the improvement in the corrosion mitigation behavior by KI.

### 3.2 Impedance measurements

EIS is a non-destructive analytical tool used to understand the adsorption of PEI-GO on the surface of metallic electrodes. The EIS technique is used in a myriad of electroanalytical applications such as electrocatalysis, chemical sensors, and corrosion.^41–45^ The EIS technique involves applying a small amplitude AC signal over a suitable frequency range and then measuring the response, which is displayed as (i) real and imaginary/complex resistance plots (Nyquist curves), (ii) logarithm of frequency *vs.* impedance modulus (Bode plot) and (iii) log frequency *vs.* phase angle (phase angle plots).^32,46^ In the presence of the corrosion inhibitor in an aggressive corrosive electrolyte, a thin protective film of PEI-GO molecules was formed on the surface of the metal surface under investigation. This resulted in an increase in the observed resistance values due to the prevention of the mass and the charge transfer processes.^47–49^

**Fig. 2:**
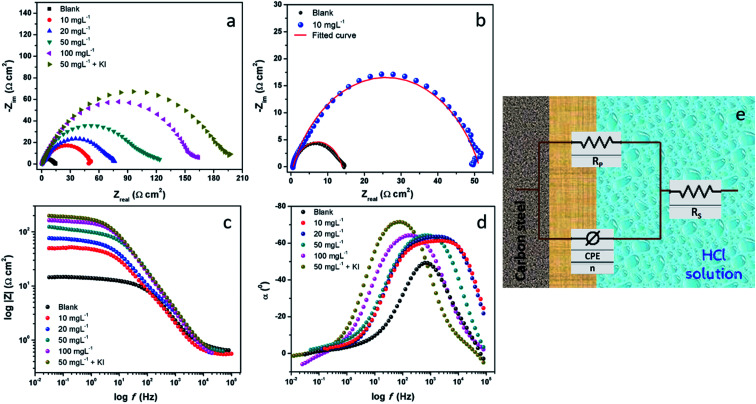
EIS curves for the carbon steel sample immersed in 15% HCl in the absence and presence of different concentrations of PEI-GO and containing KI. (a) Nyquist and (b) fitted curves, (c) Bode curves, (d) phase angle curves, and (e) electrical equivalent circuit diagram.

The result of the addition of the inhibitor PEI-GO with varying concentrations in the corrosive electrolyte is shown as Nyquist plots in Fig. 2(a). A noticeable feature observed in Nyquist plots is that semicircular arcs are obtained, wherein their center lies below the real *x*-axis. The obtained depressed semicircles are attributed to the frequency dispersion behavior due to surface heterogeneity arising on the electrode surface because of corrosion damage.^50–52^ In this case, instead of using an ideal double-layer capacitor, a constant phase element (CPE) is used to model the frequency dispersion behavior accurately. The appearance of single depressed semicircles in the Nyquist plots corresponds to a charge transfer-controlled process with one time constant. The equivalent circuit diagram, which was used to fit the EIS data, is shown in Fig. 2(e), and the fitted Nyquist curves are shown in Fig. 2(b). The circuit consists of a polarization resistance (*R*_P_) with the CPE and *R*_s_ (solution resistance). The CPE impedance is expressed as:^53–56^

5 *Z*_CPE_ = *Y*_0_^−1^(*jw*)^−*n*^

where *Y*_0_ is the proportionality constant, and the other symbols are as described earlier.^53^ The obtained electrochemical impedance parameters listed in Table 2 together with the goodness of fit parameter (*χ*^2^) show the acceptability of the proposed equivalent circuit.

**Table 2:** Electrochemical impedance parameters at varying dosages of PEI-GO without and with 5 mM KI

Inhibitor concentration (mg L^−1^)	*R*_s_ (Ω cm^2^)	*R*_p_ (Ω cm^2^)	*Y*_0_ × 10^−6^ (S × s^a^)	*n*	*χ* ^2^ × 10^−3^	*η*%
Blank	0.64	13.30 ± 1.32	592.1	0.77	2.058	—
10	1.81	51.50 ± 0.77	468.3	0.81	2.35	74.17
25	1.52	75.23 ± 0.78	384.2	0.85	1.92	82.32
50	0.94	117.4 ± 1.13	299.6	0.84	3.61	88.67
100	1.32	152.8 + 0.95	245.3	0.86	4.23	91.29
50 + 5 mM KI	1.11	191.2 ± 1.03	186.2	0.88	3.21	93.04

There was a successive increase in the polarization resistance upon the addition of increasing concentrations of the inhibitor PEI-GO. This observation corroborated the increase in the corrosion inhibition effectiveness with an increase in the concentration of the PEI-GO inhibitor in acidic electrolyte. The inhibition efficiency can be computed as:^40,53^6
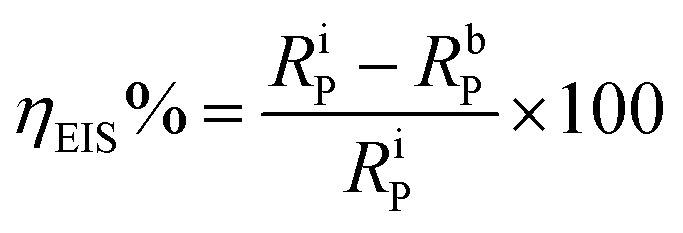
where *R*^i^_P_ and *R*^b^_P_ denote the polarization resistance without and with PEI-GO, respectively. Upon the addition of successive concentrations of the inhibitor PEI-GO, there was an increase in the *R*_P_ value from 13.30 Ω cm^2^ to 152.8 Ω cm^2^ at a concentration of 100 mg L^−1^, which is ascribed to the inhibition behavior of the inhibitor PEI-GO. This indicates that the inhibition efficiency reached a value of 91.29% in the acidic electrolyte. Upon the introduction of iodide ions in the form of potassium iodide with an increasing concentration (at 5 mM concentration), a further increase in the corrosion inhibition efficiency was observed, which reached up to 93.04%, suggesting the successful corrosion inhibition of PEI-GO.

This behavior is attributed to the adsorption of the negatively charged iodide on the metallic surface *via* electrostatic attraction. The polymeric amine groups of the functionalized GO become protonated in the corrosive acidic medium and undergo coulombic attraction with the iodide ions already adsorbed on the steel surface.^38,57–59^

The Bode and the phase angle plots are presented in Fig. 2c and d, respectively. A close look at the Bode plots reveals that there is an increase in the Bode modulus with an increase in the concentration of the inhibitor. A corresponding increase was also observed in the phase angle plots upon the addition of the PEI-GO inhibitor, which reflects an improvement in the capacitive behavior of the metal and solution interface upon the addition of increasing concentrations of inhibitor molecules.^36,60,61^ The Bode slopes and the phase angles approach −1° to 90°, indicating an enhancement in the capacitive behavior of the corrosion inhibitor.

### 3.3 Electrochemical frequency modulation

**Fig. 3:**
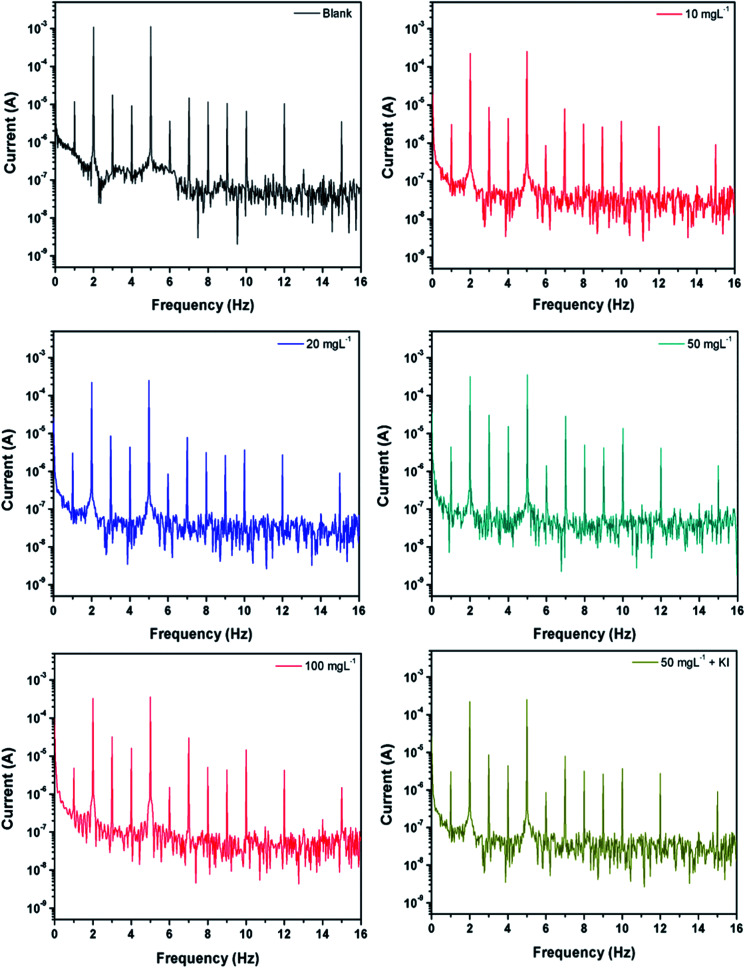
EFM spectra recorded without and with varying concentrations of PEI-GO and containing 5 mM KI.

EFM is another non-destructive technique, which takes minimal time without the need to measure the Tafel constants, and it provides the instantaneous corrosion current densities.^62,63^ An attractive feature of this technique is the use of causality factors 2 and 3 (CF2 and CF3), which provide a direct validation of the experimentally obtained results. The corrosion inhibition efficiency from the electrochemical frequency modulation was calculated as follows:^56,63,64^7
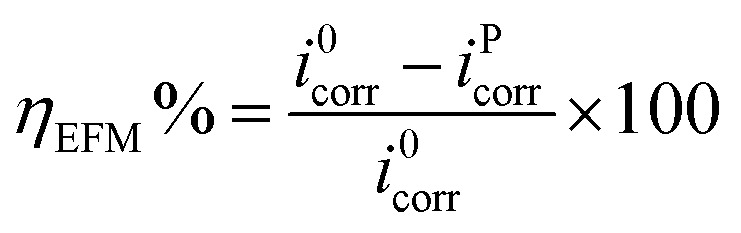
where *i*^0^_corr_ and *i*^P^_corr_ represent the corrosion current density in the presence and absence of the inhibitor PEI-GO, respectively, as observed from the EFM measurements. The obtained EFM spectra of the carbon steel surface are shown in Fig. 3, and the corresponding data is listed in Table 3. It is evident from Fig. 3 that an apparent decrease in the corrosion current density occurred upon the addition of the inhibitor PEI-GO, which supports the corrosion protection by the PEI-GO inhibitor. Besides, the values of causality factors CF2 and CF3 are in the range of 2 and 3, which are consistent with the EFM theory and verify the obtained EFM results.

**Table 3:** EFM data for the carbon steel substrate in 15% HCl in the absence and with different concentrations of PEI-GO in the presence of KI

Inhibitor (mg L^−1^)	*i*_corr_ (μA cm^−2^)	Causality factor 2	Causality factor 3	*η*%
Blank	1986.3	2.013	2.997	—
10	645	2.035	2.979	67.53
25	584	2.026	3.011	70.60
50	219	2.043	3.153	89.10
100	184	1.996	3.046	90.73
50 + KI	115	2.053	3.055	94.53

### 3.4 Polarization studies

**Fig. 4:**
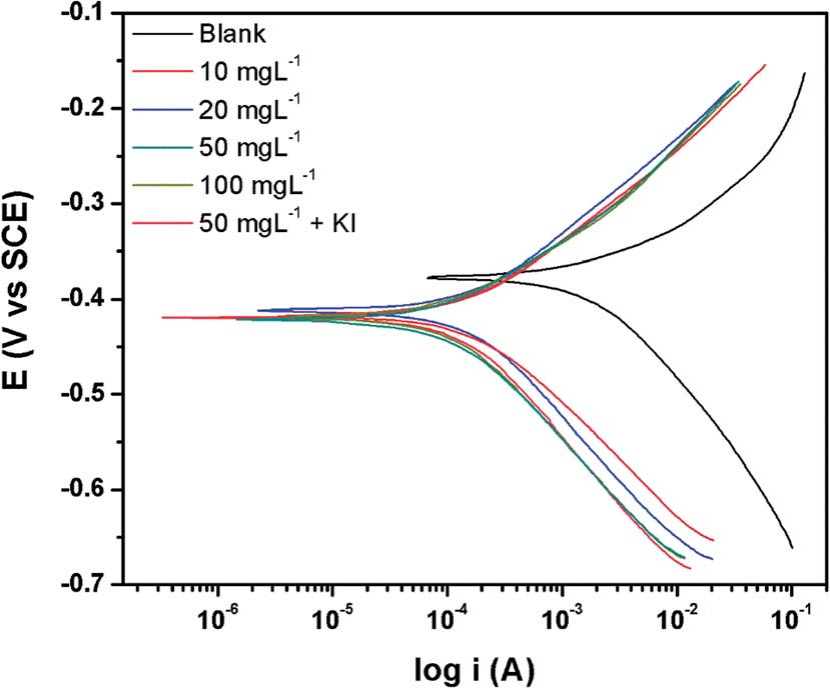
Tafel curves for the metallic electrode with the varying concentrations of PEI-GO and containing 5 mM KI.

The polarization plots in the absence and presence of the inhibitor PEI-GO with increasing concentrations and the presence of KI additive are depicted in Fig. 4. The electrochemical corrosion parameters and the inhibition efficiency (*η*_PDP_%) were evaluated by extrapolation of the linear segments of the anodic/cathodic Tafel slope and presented in Table 4. The corrosion inhibition efficiency was calculated as follows:^21,36,40^8
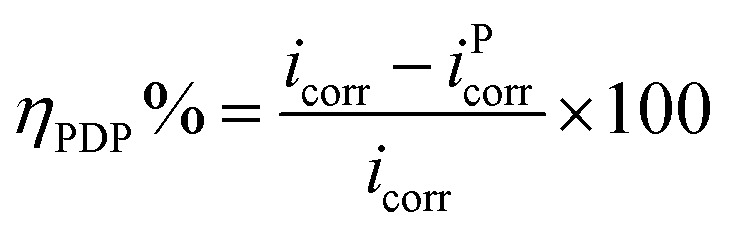
where *i*_corr_ and *i*^P^_corr_ denote the corrosion current densities, as mentioned above. The addition of the PEI-GO corrosion inhibitor with increasing concentrations leads to a significant decline in the anodic/cathodic corrosion current densities. This indicates the suppression of both the anodic electrodissolution of iron and cathodic hydrogen evolution. However, there was no change in the slope of the current curves, which suggests that the addition of the inhibitor PEI-GO did not produce a different mechanism of corrosion. A shift in the corrosion potential (*E*_corr_) values towards the cathodic direction was observed with the addition of the inhibitor PEI-GO, which, however, was not much prominent and indicates the inclination of the inhibitor towards the mitigation of corrosion with cathodic predominance.^21,53,65,66^

**Table 4:** Potentiodynamic polarization parameters for the steel substrate at varying concentrations of PEI-GO and with KI

Inhibitor concentration (mg L^−1^)	*i*_corr_ (μA cm^−2^)	−*E*_corr_ (mV per SCE)	*β*_a_ (mV dec^−1^)	−*β*_c_ (mV dec^−1^)	*η*_PDP_%
Blank	1127.3	378	67.1	141.1	—
10	467.3	413	73.1	156.3	58.57
25	323.1	418	77.4	162.4	71.35
50	160.5	420	69.3	166.1	85.80
100	125.2	422	81.1	161.4	88.91
50 + KI	90.9	424	75.3	172.4	92.02

The introduction of PEI-GO in the corrosive acidic electrolyte of 15% HCl produced a considerable decline in the corrosion current density from 1127.3 μA cm^−2^ to 125.2 μA cm^−2^, which further decreased to 90.9 μA cm^−2^ in the presence of KI. This behavior reflects the effective inhibition of Fe dissolution and the hydrogen evolution reaction.^61,67–69^ Besides, both the cathodic and anodic Tafel branches of the Tafel graphs indicate a noticeable shift in the current density towards lower values in the presence of PEI-GO, which means PEI-GO exhibits a mixed-type inhibitor action. The introduction of increasing concentrations of PEI-GO to the acid solution resulted in an increase in corrosion inhibition owing to the adsorption of the corrosion inhibitor on the metal surface. Subsequently, the addition of iodide ions to the corrosive acidic solution further decreased the corrosion current density, which supports an improvement in the corrosion inhibiting action of PEI-GO.

### 3.5 Surface analysis

**Fig. 5:**
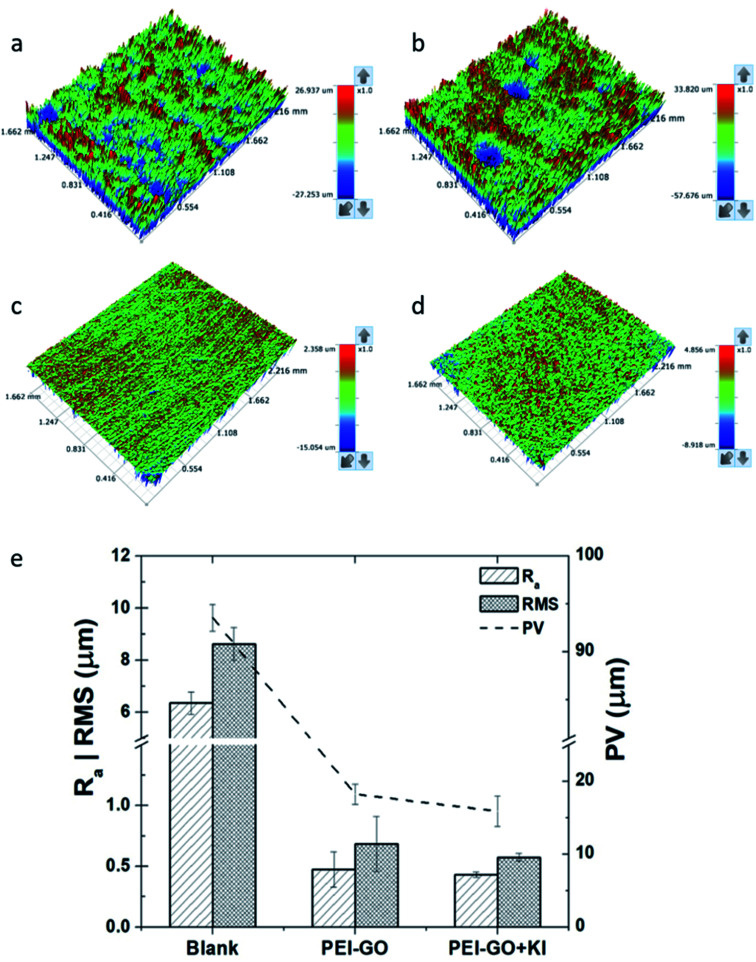
Typical 3D optical profilometry images recorded after immersion in 15% HCl showing the surface topography of carbon steel (a) blank (without pits), (b) blank (with pits), (c) with PEI-GO and (d) with PEI-GO with KI samples, (e) surface roughness parameters of the blank, PEI-GO and PEI-GO with KI samples.

The 3D optical profilometry images of the samples after immersion in the blank corrosive electrolyte, electrolyte with PEI-GO, and PEI-GO with KI inhibitor for 24 h are shown in Fig. 5(a). The influence of the inhibitor can be observed on the surface topography of the samples after 24 h. The samples immersed in the blank possess a highly rough surface texture, as shown in Fig. 5(a), and the influence of the inhibitor on the surface texture of the carbon steel samples can be clearly observed. The samples immersed in acidic medium with the PEI-GO inhibitor exhibited a smoother surface texture, while the addition of KI resulted in a better surface profile. The surface roughness parameters obtained are presented in Fig. 5(b), and it can be observed that the surface of the steel samples showed considerable smoothness in the presence of the inhibitor PEI-GO. *R*_a_, which is the mean amplitude roughness parameter, was significantly reduced by about 92%. Furthermore, the root-mean-square (RMS) and the maximum depth to minimum valley distance (PV) decreased at a similar proportion. This supports the formation of an inhibitive and protective film on the metallic substrate, hindering the dissolution of ions. It was also observed that the blank steel sample exhibited a considerable increase in the number of pits, as shown in Fig. 5(c). The pits were randomly distributed on the steel surface, and the maximal depth of the pits measured using the 3D profilometer was in the range of 55 ± 5 μm. Further, upon the addition of iodide ions in the form of KI, a smoother surface was obtained for the steel sample (see Fig. 5(d)). The corresponding reduction in the *R*_a_, RMS, and PV roughness parameters of the metallic specimens with the introduction of KI was 93.2%, 93.3%, and 83%, respectively. The considerable improvement in the steel surface smoothness in the presence of the corrosion inhibitor PEI-GO and further upon the addition of KI supports the formation of a smooth inhibitive and protective film.

**Fig. 6:**
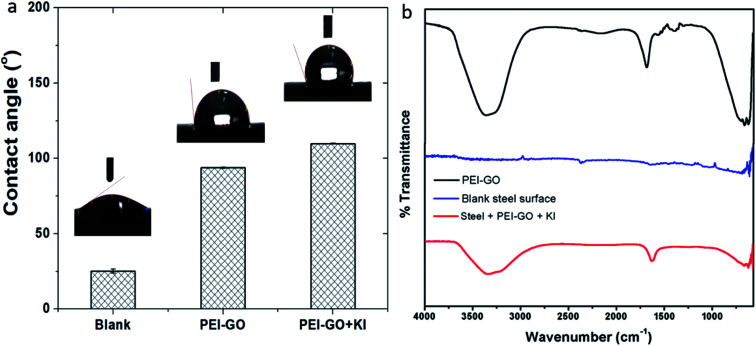
(a) Water contact angles of the blank, PEI-GO and PEI-GO with KI samples and (b) FTIR results obtained for the steel surface following immersion in the corrosive solution without and with PEI-GO + KI.

Contact angle measurements were performed to understand the influence of the adsorbed PEI-GO film on the hydrophobicity of the steel specimens, and the results are shown in Fig. 6(a). Compared to the blank sample with a contact angle of 25 ± 1.3°, there was a significant increase in the contact angle value upon the addition of the inhibitor PEI-GO. It has been reported that inhibitors possessing long aliphatic tails are hydrophobic in nature.^70^ This suggests that the steel surface became more hydrophobic with the addition of PEI-GO. Similarly, the introduction of iodide ions further increased the contact angle by modifying the surface to exhibit more hydrophobic characteristics. This can be associated with the formation of the inhibitor film with distinctive functional groups.

The adsorption of PEI-GO with KI on the carbon steel surface was evaluated *via* FTIR-ATR measurements. The steel specimens were immersed without and with the optimum concentration of the inhibitor, and their FTIR spectra were recorded after 24 h, as shown in Fig. 6(b). In the pure PEI-GO, the band at around 1650 cm^−1^ is assigned to –C=O, the band at 1620 cm^−1^ corresponds to the aromatic –C=C–, and the two bands in the range of 3000 to 3500 cm^−1^ are attributed to the N–H stretch (asymmetric and symmetric). The FTIR spectra of the inhibited samples exhibit similar characteristics as that of the pure PEI-GO samples, supporting the adsorption of the inhibitor. Furthermore, the low peak intensities for the steel specimens indicate the availability of trace amounts of inhibitor on the steel surface, which corresponds to a thin inhibitor film. This observation is in accordance with the earlier observed results by Umoren *et al.* and Priyanka *et al.*^21,37,38^

### 3.6 Quantum chemical study

**Fig. 7:**
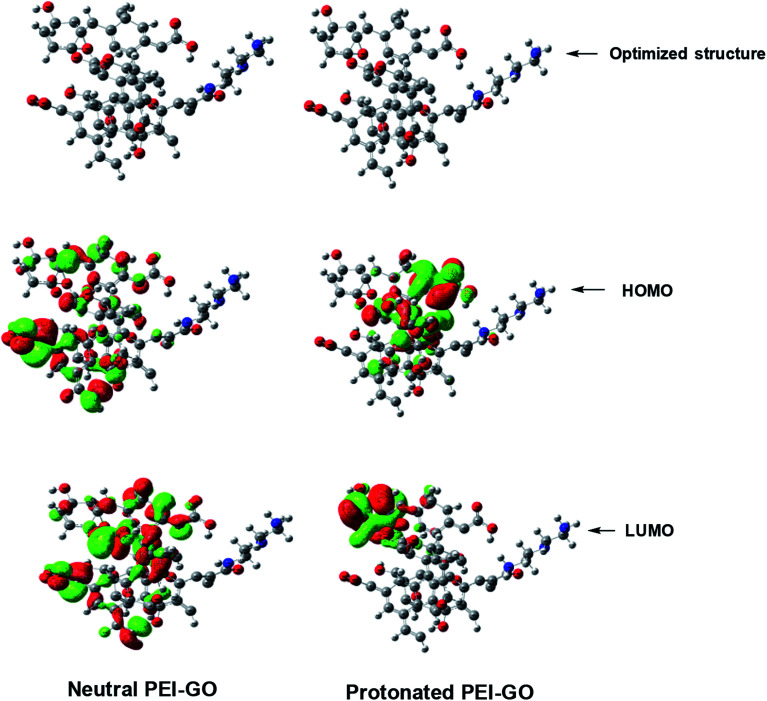
Optimized molecular structures and the HOMO and LUMO electron density distributions of the neutral and protonated PEI-GO.

The optimized geometry, *E*_HOMO_, and *E*_LUMO_ of GO, neutral PEI-GO, and protonated PEI-GO are shown in Fig. 7. According to the frontier molecular orbital theory, only the frontier molecular orbitals, *i.e.*, the HOMO and LUMO, are involved in the interaction of the inhibitor molecules with metal surface.^20^ In general, the higher the HOMO value, the higher the electron-donating capacity of the inhibitor to the vacant d-orbital of the metal. In contrast, the lower the LUMO value, the greater the electron-accepting ability of the inhibitor from the filled metal orbitals. However, the most important parameter is Δ*E*, which is the energy difference between the LUMO and HOMO. The lower the Δ*E* value, the easier is the release of an electron, and the stronger would be the adsorption.^56^ Thus, a good corrosion inhibitor should not only have the ability to donate an electron, but also to accept it in its vacant orbitals.^71^

Inspection of Table 5 reveals that the *E*_HOMO_ values of neutral PEI-GO is greater than that of protonated PEI-GO, and thus the neutral PEI-GO has more electron-donating capacity than protonated PEI-GO. However, the *E*_LUMO_ value of neutral PEI-GO is lower than that of protonated PEI-GO, which makes neutral PEI-GO accept more electrons than protonated PEI-GO from the filled d-orbital of steel, resulting in its stronger adsorption. Additionally, the Δ*E* value of neutral PEI-GO is lower than that of protonated PEI-GO, which causes neutral PEI-GO to release electrons easily, consequently strengthening its adsorption.

**Table 5:** Calculated quantum chemical parameters

System	*E*_HOMO_ (eV)	*E*_LUMO_ (eV)	Δ*E* (eV)
PEI-GO (neutral)	−4.846	−4.209	0.637
PEI-GO (protonated)	−4.940	−3.328	1.612

### 3.7 Comparison with earlier reports

As stated above, the present investigation is the first application of chemically functionalized GO against the carbon steel corrosion in acidizing conditions, which presents a complex and aggressive medium. A comparison of PEI-GO with the earlier reported heterocyclic compound-based inhibitors is presented in Table 6. The majority of the previous reported inhibitors are based on nitrogen-based multiple aromatic ring-containing molecules such as pyridines, pyrazolones, and naphthyridines, as presented in Table 6.^24,25,72–78^ On the other hand, the present inhibitor, PEI-GO, is a simple aliphatic chain-containing polymeric molecule, showing excellent solubility in acid solution. Therefore, it exhibits a higher corrosion inhibition efficiency at a lower required dosage compared to the heterocyclic compound-based corrosion inhibitors.

**Table 6:** Comparative chart showing the performance of the inhibitors based on heterocyclic compounds and inhibitors based on nanomaterials

Type	Inhibitor name	Metal/alloy; corrosive medium	Optimum concentration of inhibitor	*η* (%)	Reference
Heterocylic compound-based inhibitors	Pyrimidine derivatives	N80 steel; 20% H_2_SO_4_	250 mg L^−1^	89.10	24
	Pyridine derivatives	N80 steel; 15% HCl	200 mg L^−1^	90.24	25
	Pyrazolone derivatives	N80 steel; 15% HCl	200 mg L^−1^	93.90	72
	Naphthyridine derivatives	N80 steel; 15% HCl	150 mg L^−1^	93.90	73
	Isatin derivatives	Mild steel; 20% H_2_SO_4_	300 mg L^−1^	97.20	74
	Schiff bases of isatin	Mild steel; 20% H_2_SO_4_	200 mg L^−1^	98.60	75
	Pyran derivatives	N80 steel; 15% HCl	300 mg L^−1^	97.70	76
	Chromenopyrazol	N80 steel; 15% HCl	400 mg L^−1^	98.40	77
Nanomaterial-based inhibitors	Diaminopyridine-GO	Mild steel; 1 M HCl	25 mg L^−1^	95.08	9
	Diazopyridine-GO		25 mg L^−1^	96.73	
	*p*-Aminophenol-GO	Mild steel; 1 M HCl	25 mg L^−1^	92.86	10
	Aminoazobenzene-GO	Mild steel; 1 M HCl	25 mg L^−1^	96.80	11
	Diaminobenzene-GO		25 mg L^−1^	95.20	
	Chitosan-AgNPs	St37 steel; 15% HCl	1000 mg L^−1^	84.68	39
	Chitosan-AgNPs	St37 steel; 15% H_2_SO_4_	1000 mg L^−1^	94.98	80
	Polyethyleneglycol-ZnO	Mild steel; 5% HCl	1000 mg L^−1^	64.08	81
	Polyvinylpyrrolidone-ZnO		1000 mg L^−1^	70.39	
	Polyacrylonitrile-ZnO		1000 mg L^−1^	79.91	
	Dextran-AgNPs	St37-2 steel; 15% H_2_SO_4_	1000 mg L^−1^	89.47	82
	PEI-GO	Carbon steel; 15% HCl	50 mg L^−1^	88.24	Present work
	PEI-GO + KI		50 mg L^−1^ + 5 mM	95.77	

A comparison of the efficiency of nanomaterial-based inhibitors used in acid solutions is also displayed in Table 6. Preliminary inspection of the table reveals that only three studies are available on modified GO as a promising inhibitor in 1 M HCl media.^9–11^ The other studies include polymer–AgNP and polymer–ZnO composites as inhibitors for the corrosion of steel in acidizing conditions. Therefore, it is evident that the present study is the first available report on the application of chemically modified GO on oil-well acidizing conditions. Another remarkable merit associated with the current work is that the optimum inhibitor concentration is 50 mg L^−1^, which is much lower compared to that in the previous reports.^39,79–82^

### 3.8 Mechanism of adsorption and inhibition

The weight loss measurements show that the inhibition efficiency increased with an increase in the inhibitor concentration. The EIS studies show that the polarization resistance increased with an increase in inhibitor concentration. This suggests that the inhibitor molecules act by adsorption on the steel surface, and that the organic inhibitor molecules replace the water molecules that are pre-adsorbed at the metal/electrolyte interface. This lowers the local dielectric constant, which results in an improvement in the capacitive performance of the metallic surface. The increase in the polarization resistance with inhibitor concentration supports the fact that the inhibitor adsorption on the steel surface creates a barrier in the charge transfer process during corrosion. These results are also consistent in terms of an increase in the energy of activation, which was observed in the influence of temperature study. According to the PDP analysis, PEI-GO acts as a mixed-type inhibitor. Thus, the mechanism of adsorption of PEI-GO on the metal surface is as follows. Firstly, the neutral inhibitor molecules adsorb on the metal steel surface *via* a chemisorption mechanism by sharing their lone pairs of electrons, which are present on O and N atom with the Fe atoms. Also, the inhibitor molecules can be adsorbed *via* the chemisorption mechanism using the π-electrons of their benzene rings and the vacant d-orbitals of iron. Secondly, the protonated inhibitor molecules may be physically adsorbed by the electrostatic interactions between the protonated sites and the pre-adsorbed halide ions. The third adsorption mode includes the electron transfer from the filled metal d-orbital to the vacant anti-bonding orbitals of the inhibitor molecules (retro-donation), which strengthens the adsorption.

## 4 Conclusions

The corrosion inhibition behavior of polyethyleneimine-modified graphene oxide (PEI-GO) was investigated on the surface of carbon steel in 15% HCl solution. The corrosion inhibition evaluation was carried out using the weight-loss method and electrochemical techniques (EIS, EFM, and PDP), and supported with surface analysis *via* water contact angle measurements, 3-D profilometry, and FTIR-ATR spectroscopy. The conclusions drawn are as follows:

1. The corrosion inhibition efficiency improved with an increase in the inhibitor concentration and reached 88.24%, which was further enhanced up to 96.67% upon the addition of KI.

2. The adsorption of PEI-GO on the steel surface followed the Langmuir adsorption model.

3. The EIS measurements revealed an increase in the polarization resistance, which supports the formation of the inhibitor film.

4. The EFM and PDP measurements showed a decline in the corrosion currents, wherein the inhibitor exhibited predominantly cathodic inhibition behavior.

5. The surface studies of the inhibitor film using water contact angle measurements and 3D-profilometry showed an improvement in the hydrophobicity and surface smoothness, supporting the formation of a smooth inhibitor film on the steel surface.

6. The FTIR-ATR studies revealed the existence of the PEI-GO functional groups on the steel surface, which again suggests the formation of the inhibitor film.

7. The DFT-based computational studies show that the inhibitor acts in both the neutral and protonated forms.

## Author statement

All the authors have read the manuscript and agree with the submission.

## Conflicts of interest

The authors declare no conflict of interest.

## Supplementary Material
